# Stepwise PathNet: a layer-by-layer knowledge-selection-based transfer learning algorithm

**DOI:** 10.1038/s41598-020-64165-3

**Published:** 2020-05-18

**Authors:** Shunsuke Imai, Shin Kawai, Hajime Nobuhara

**Affiliations:** Department of Intelligent Interaction Technologies, Graduate School of Systems and Information Engineering, University of Tsukuba, 1-1-1 Tennoudai, Tsukuba, Ibaraki 305-8573 Japan

**Keywords:** Computational neuroscience, Engineering

## Abstract

Some neural network can be trained by transfer learning, which uses a pre-trained neural network as the source task, for a small target task’s dataset. The performance of the transfer learning depends on the knowledge (i.e., layers) selected from the pre-trained network. At present, this knowledge is usually chosen by humans. The transfer learning method PathNet automatically selects pre-trained modules or adjustable modules in a modular neural network. However, PathNet requires modular neural networks as the pre-trained networks, therefore non-modular pre-trained neural networks are currently unavailable. Consequently, PathNet limits the versatility of the network structure. To address this limitation, we propose Stepwise PathNet, which regards the layers of a non-modular pre-trained neural network as the module in PathNet and selects the layers automatically through training. In an experimental validation of transfer learning from InceptionV3 pre-trained on the ImageNet dataset to networks trained on three other datasets (CIFAR-100, SVHN and Food-101), Stepwise PathNet was up to 8% and 10% more accurate than finely tuned and from-scratch approaches, respectively. Also, some of the selected layers were not supported by the layer functions assumed in PathNet.

## Introduction

A neural network is a machine learning method, and it requires a relatively large labeled training dataset. This requirement has been met by transfer learning^1^. For example, a large dataset of labeled photographs both with and without cats are needed to train a neural network that recognizes cats in photographs. When the task involves rare animals, it may by hard to obtain a sufficiently large training dataset. Transfer learning reduces the required size of the training dataset for the target task, which addresses this problem. To this end, it exploits the knowledge gained by a pre-trained neural network. Learning by the pre-trained neural network (called the source task) constitutes the first learning task of the transfer learning. The training dataset is then reduced in size for the second learning task (i.e., the target task).

Some machine learning methods must be appropriately initialized to ensure their high performance. In pre-training on deep belief networks^2^ and self-taught learning^3^, the initial parameters are obtained by unsupervised learning. Similarly, the performance of a convolutional neural network (CNN) can be improved by fine-tuning the initial pre-trained parameters^4,5^. Transfer learning is efficient when the target task has a scarce dataset^6^, but can actually decrease the performance of a pre-trained CNN. Such a performance decline is called “negative transfer”^7^. After fixing its parameters, the pre-trained layer of a CNN behaves as a feature extractor. Reportedly, increasing the number of adjustable layers to be learned (including learning by fine-tuning) associates an excessive number of parameters with the dataset, leading to the well-known overfitting problem. Moreover, the performance depends on the positions and number of layers to be fixed^8^. Fixing-based methods are expected to avoid the overfitting problem. When the training dataset of the target task is scarce, overfitting caused by an excessive number of parameters (i.e. an overly complex model) can be regularized using a joint Bayesian method for face verification (rather than a CNN) for transfer learning^9^.

Interpreting and understanding neural networks is important for transfer learning^10^. A CNN extracts the low-dimensional information (e.g., color and edges) in its bottom layer, and the higher-dimensional (i.e., label-specific) information in its top layer^11^. The parameters learned in the bottom layer are often used for transfer learning. However, a transfer learning approach that learns the first plural convolution layers and the last fully connected layer, while fixing all other layers, proved the most effective learning technique for Bengali numeral classification (NumtaDB) in the VGG16^12^ architecture pre-trained on the ImageNet database^13,14^. Therefore, during transfer learning of a CNN, selection supported by the function of the layer is unlikely to be the most effective selection method.

**Figure 1:**
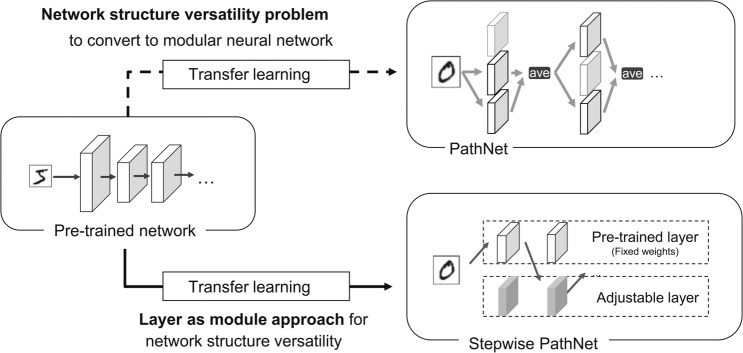
Comparison between PathNet and Stepwise Pathnet.

A method that automatically chooses the pre-trained CNNs has been proposed^7^, but this method does not perform layer-by-layer selection. PathNet^15^ is a transfer learning method that automatically selects small layers (modules) in the neural network (top left of Fig. 1). In PathNet, the selections from the fixed pre-trained modules and the adjustable modules in the transfer learning on modular neural networks are optimized by a tournament selection algorithm (TSA) based on a microbiological genetic algorithm^16^. A modular neural network contains a layer of multiple modules (small layers that may be convolutional, fully connected, or residual). In each layer, a subset of the modules in the layer is selected for learning and inference. The TSA optimizes this module selection by (i) maximizing the accuracy of the training data and (ii) training the adjustable modules using a normal neural-network optimizer [e.g., stochastic gradient descent (SGD)]. In this way, PathNet can automatically select the pre-trained knowledge as modules during transfer learning. In the modular neural network, which PathNet’s TSA deals with, one layer consists of multiple paralleled modules. In other words, the modular neural network can be considered the particular case of the general neural networks whose layers are divided into multiple small layers (i.e., modules). Therefore, pre-trained neural networks which PathNet uses must be a modular neural network, and a non-modular CNN is hard to be used even if the module supports a convolutional layer. The current PathNet is available for modular neural networks only, and needs to be extended to general neural network structures (such as CNNs).

We proposed Stepwise PathNet^17^, an extension of PathNet on the purpose of using CNNs and other non-modular neural networks. Stepwise PathNet achieves the purpose by regarding layers as modules (bottom of Fig. 1). During transfer learning, the original PathNet uses TSA to select multiple modules from each layer of the pre-trained modular neural network. This constructs the same number of the layer with the pre-trained network, but each layer-shape will differ. Our Stepwise PathNet selects a pre-trained (fixed-parameter) layer or an adjustable layer at each layer during transfer learning so that the TSA can construct the same architecture of the pre-trained neural networks. In Stepwise PathNet, TSA treats a layer as a module, i.e., every two types of layers are the same layer-shape from the pre-trained network. The TSA optimizes selecting them for each layer to construct the same architecture of the pre-trained neural networks. Moreover, the modified TSA treats this layer as a module; that is, one layer must always be selected from one of two types of layers (pre-trained or adjustable). Therefore, Stepwise PathNet exploits PathNet’s selecting the pieces of knowledges in the layer to select them on layer-by-layer. The present experiment evaluates transfer learning to CIFAR-100^18^ from Inception V3^19^ pre-trained on ImageNet^13^. The effects of modifying the TSA (i.e., accelerating and stabilizing the learning curve) are assessed, and the accuracy, speed, and stability of the learning are compared between (i) random and pre-trained initial values and (ii) fine-tuning and from-scratch without transfer learning. The main contributions and novelty of this work are summarized below.

- The presented transfer learning algorithm, which based on layer-by-layer selection and an evolutionary computation, is applicable to huge complex models in recent deep learning and the neural network field.
- The relations between layer selection and transfer-learning performance on CNNs are determined.

## Results

### Experimental Conditions

**Figure 2:**
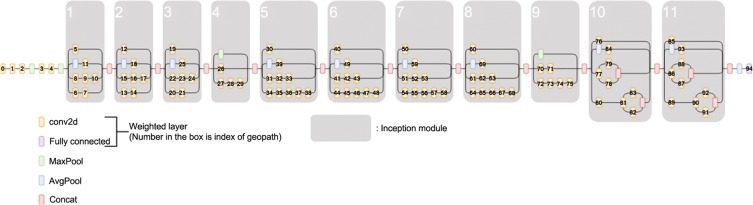
InceptionV3 model (the numbers in the conv2d and Fully Connected layers refer to the geopath indices, and the gray areas indicate the individual inception modules).

The transfer-learning performance of Stepwise PathNet using a CNN was evaluated on three datasets under InceptionV3^19^ (see Fig. 2) pre-trained to ImageNet.

#### Model architecture

InceptionV3 is an upgraded version of GoogLeNet^20^, which won the Imagenet Large Scale Visual Recognition Challenge in 2014 (ILSVRC2014). InceptionV3 is a popular pre-training model for transfer learning. It contains 154 layers, including 95 weighted (convolutional and fully connected) layers. In the present experiment, InceptionV3 was pre-trained on ImageNet. This massive, general object-recognition dataset contains 1,000 classes and over one million images, and is used in the ILSVRCs.

#### Dataset and augmentation

The datasets used in the evaluation are as follows:

- CIFAR-100^18^: A 100-class general object-recognition dataset with 500 + 100 images (training + test) in each class.
- SVHN^21^: A 10-class dataset for digit recognition in real images with 73,257 + 26,032 images (training + test) in each class.
- Food-101^22^: A 101-class food-recognition dataset with 750 + 250 images (training + test) in each class.
  All images in the CIFAR-100, SVHN, and Food-101 datasets were refitted to the input size of InceptionV3. To this end, they were resized to 224 × 224 by the bilinear method. The following augmentations were applied in all cases:
- random rotation in $[-15,15]\,\deg$
- width and height shifts in $[\,-\,\mathrm{10,10] \% }$
- horizontal flipping.

These augmentations were applied in real-time when the images were loaded in the training process (i.e., loaded to the batch).

#### Evaluations in the present experiment

The present experiment performed three evaluations: (i) a comparison among the TSAs, (ii) a comparison of Stepwise PathNet and other learning algorithms, and (iii) an evaluation of the layer selection. In the first evaluation, we compared

- “proposal 1”: Stepwise PathNet with PathNet’s original TSA,
- “proposal 2”: Stepwise PathNet with the modified TSA,
- “proposal 3”: Stepwise PathNet with the modified TSA + pre-trained initialization.
  The adjustable layers were initialized using pre-trained weights in modified TSA + pre-trained initialization (“proposal 3”), and using random variables in the original and modified TSAs (“proposal 1” and “proposal 2” respectively). In the second evaluation, we compared Stepwise PathNet with
- “conventional 1”: from scratch,
- “conventional 2”: fine-tuning.
  We also compared Stepwise PathNet with modified TSA + pre-trained initialization (“proposal 3”). Fine-tuning is a transfer learning method that uses the pre-trained weights (except those in the top layers) as initial parameters. Therefore, in the present experiment, we replaced the top 94th layer of Inception V3 (a 1,000-node fully connected layer) by a 100-node fully connected adjustable layer, and initialized it with random variables. All other layers were initialized with parameters that were pre-trained on ImageNet. From-scratch means that all parameters in InceptionV3 were initialized randomly, with no transfer learning. Note that in one epoch of fine-tuning and from-scratch, the training dataset was scanned once, whereas in one generation of Stepwise PathNet, it was scanned twice. For this reason, the x-axis of the learning curve was labeled not as “epoch”, but as “number of scanned datasets”. All algorithms were optimized by Adam^23^ with the Keras default parameters^24^. Each algorithm was iterated up to 60 scans of the dataset (i.e., 60 epochs in fine-tuning and from-scratch, and 30 generations in Stepwise PathNet). Also, each algorithm was executed on a Geforce GTX1080Ti graphics card with a batch size of 16. In all cases, the Stepwise PathNet parameters were set as follows:
- Number of geopaths: 20 (unchanged from the original PathNet)
- Length of geopath: 95 (number of weighted layers)
- Probability of mutation: $\frac{1}{95}$.

The third evaluation was a heatmap evaluation of the layer selection on 10 learning samples selected from the three datasets.

### Comparison of TSAs

Table 1 presents the results of all algorithms on the three datasets. On average, proposal 2 (with random initialization) was up to 15.8% more accurate than proposal 1 (with random initialization), but its accuracy dropped by 0.1% on SVHN. Proposal 3 outperformed proposal 2 in all cases, indicating a positive effect of the pre-trained initialization. Also, proposal 3 was 20.8% more accurate (on average) than proposal 1 on CIFAR-100. The improvements of average test accuracy in proposal 3 over that of proposal 1 were ranked as follows: CIFAR-100 (+20.8%) > Food-101 (+13.2%) > SVHN (+1%).

**Table 1:** Results of the compared algorithms and datasets.

		CIFAR-100				SVHN				Food-101			
		loss	train accuracy [%]	loss	test accuracy [%]	loss	train accuracy [%]	loss	test accuracy [%]	loss	train accuracy [%]	loss	test accuracy [%]
Stepwise PathNet with unmodified TSA (proposed 1)	0	1.398	60.3	1.499	62.5	0.028	99.1	0.244	**95**.**6**	1.433	61.6	2.359	52.0
	1	**0**.**632**	**80**.**5**	1.197	**70**.**6**	0.024	99.2	0.264	95.3	0.287	91.2	2.532	59.1
	2	1.624	54.9	1.893	56.6	0.02	**99**.**4**	0.262	95.5	0.246	92.3	2.929	57.1
	3	1.226	64.8	1.763	61.1	**0**.**019**	**99**.**4**	0.288	94.6	0.308	90.8	2.953	52.8
	4	0.846	74.8	**1.116**	69.8	0.029	99.0	0.265	95.2	**0.143**	**95.9**	2.663	**59.3**
	5	1.166	66.2	1.497	60.5	0.022	**99.4**	0.318	95.3	2.517	37.4	2.057	46.8
	6	2.461	36.5	2.820	34.4	0.023	99.3	0.274	95.4	0.737	78.6	2.438	53.6
	7	2.551	50.8	3.055	46.2	0.075	97.7	0.242	94.9	0.236	93.2	2.803	53.6
	8	1.285	63.2	1.396	61.7	0.030	99.1	**0.238**	95.5	0.944	73.9	**1.917**	57.3
	9	0.88	73.9	1.144	69.4	0.019	**99.4**	0.292	95.1	0.162	95.1	3.082	53.4
	Ave.	1.465	61.3	1.804	58.2	0.030	99.1	0.266	95.3	0.762	79.4	2.517	54.6
Stepwise PathNet with modified TSA (proposed 2)	0	0.658	79.6	1.429	68.9	**0.013**	99.5	0.328	95.2	0.112	96.5	3.587	52.2
	1	0.357	88.6	**0.836**	**79.7**	**0.013**	**99.6**	0.29	**95.7**	0.081	97.5	2.620	63.7
	2	0.605	81.0	1.276	70.3	0.018	99.5	0.305	94.9	0.08	97.7	3.080	56.8
	3	**0.266**	**91.4**	1.054	78.3	0.018	99.4	**0.283**	95.5	0.065	97.9	**2.457**	66.0
	4	0.681	79.0	1.194	71.6	0.018	99.4	0.501	93.9	0.076	97.6	3.065	60.3
	5	0.513	83.8	0.989	76.0	0.015	**99.6**	0.297	95.6	0.078	97.6	2.560	**66.7**
	6	0.375	88.0	1.092	75.6	0.014	**99.6**	0.295	95.5	**0.063**	**98.0**	2.789	65.1
	7	0.338	88.9	0.863	78.8	**0.013**	**99.6**	0.308	95.6	0.077	97.5	3.123	57.9
	8	0.738	77.4	1.335	66.9	0.015	**99.6**	0.299	95.2	0.149	95.3	3.549	52.1
	9	0.96	71.5	1.643	64.9	0.015	**99.6**	0.302	95.5	0.069	97.9	3.054	61.3
	Ave.	0.503	84.2	1.119	74.0	0.015	99.5	0.323	95.2	0.087	97.3	2.981	60.1
Stepwise PathNet with modified TSA + pre-trained initialization (proposed 3)	0	**0.186**	**93.9**	0.962	81.1	0.009	99.7	0.251	96.3	**0.044**	**98.6**	2.131	71.6
	1	0.272	91.0	1.178	76.5	0.011	99.6	0.257	96.3	0.057	98.2	**2.048**	**72.1**
	2	0.230	92.3	0.986	80.9	0.014	99.6	0.269	96.2	0.083	97.4	2.178	67.6
	3	0.326	89.3	1.051	76.8	0.009	**99.7**	0.295	96.0	0.054	98.4	2.580	66.7
	4	0.262	91.4	0.934	80.8	0.011	**99.7**	0.256	96.4	0.066	97.9	2.374	68.8
	5	0.188	93.7	0.907	81.1	0.012	99.6	0.235	96.2	0.075	97.6	2.238	69.2
	6	0.235	92.3	1.077	77.9	0.013	**99.7**	0.304	95.9	0.097	97.0	2.804	62.4
	7	0.334	89.1	1.082	76.2	0.012	**99.7**	0.240	96.6	0.087	97.3	2.510	64.5
	8	0.233	92.3	0.994	79.3	**0.009**	**99.7**	**0.234**	**96.8**	0.059	98.1	2.478	69.5
	9	0.286	90.6	0.927	79.5	0.010	**99.7**	0.258	96.4	0.057	98.3	2.424	66.6
	Ave.	0.252	91.7	1.019	79.0	0.011	99.7	0.260	96.3	0.069	97.8	2.371	68.0
From scratch (conventional 1)	0	0.092	97.1	2.23	63.9	**0.048**	**98.4**	**0.193**	**96.1**	0.204	93.5	1.834	66.9
	1	**0.056**	**98.2**	2.038	70.2	0.061	98.1	0.329	94.3	0.182	94.1	**1.553**	**71.2**
	2	0.059	98.1	1.949	69.7	0.052	98.3	0.236	95.4	0.194	93.8	2.016	64.6
	3	0.074	97.7	1.940	67.6	0.054	98.2	0.240	95.3	0.178	94.3	1.884	67.1
	4	0.064	97.9	**1.895**	70.1	0.054	98.3	0.207	95.4	0.204	93.5	1.782	66.9
	5	0.062	98.0	2.114	67.6	0.058	98.2	0.200	95.8	0.179	94.2	1.801	67.3
	6	0.056	98.2	1.988	70.3	0.051	**98.4**	0.301	94.8	0.184	94.1	1.843	67.6
	7	0.067	97.8	1.902	69.6	0.063	98.1	0.251	95.1	**0.175**	**94.4**	1.697	68.7
	8	0.058	98.1	1.970	69.8	0.059	98.1	0.225	95.3	0.181	94.2	1.680	69.3
	9	0.059	98.1	1.958	**71.1**	0.060	98.1	0.196	95.8	0.182	94.1	1.934	66.1
	Ave.	0.065	97.9	2.003	68.7	0.056	98.2	0.242	95.3	0.187	94.0	1.788	67.7
Fine-tuning (conventional 2)	0	0.062	**98.0**	1.763	**73.4**	0.021	99.3	0.254	95.5	0.247	92.1	**1.557**	**70.5**
	1	0.078	97.5	1.897	71.0	**0.020**	99.3	0.220	96.1	0.244	92.2	1.891	66.8
	2	**0.061**	**98.0**	1.778	73.1	0.023	99.2	0.233	95.8	0.205	93.5	1.997	67.2
	3	0.077	97.5	2.157	68.5	**0.020**	**99.4**	0.215	**96.3**	0.252	91.8	1.963	65.7
	4	0.073	97.6	1.802	72.7	0.021	99.3	0.237	95.9	0.223	92.9	1.784	68.1
	5	0.075	97.5	1.847	72.2	**0.020**	99.3	0.227	96.0	0.209	93.4	1.892	67.6
	6	0.069	97.7	**1.749**	73.3	**0.020**	**99.4**	**0.211**	96.1	**0.197**	**93.7**	1.706	69.5
	7	0.069	97.8	1.815	72.4	0.023	99.3	0.218	96.0	0.216	93.0	1.890	67.4
	8	0.065	97.8	2.132	69.0	0.024	99.3	0.224	96.0	0.235	92.6	1.797	67.2
	9	0.084	97.3	2.757	64.2	0.021	99.3	0.213	96.2	0.206	93.5	1.758	69.1
	Ave.	0.070	97.7	1.882	71.7	0.021	99.3	0.226	96.0	0.225	92.8	1.831	67.8

Below we summarize the differences between the training and test accuracies on the CIFAR-100, SVHN, and Food-101 datasets, respectively:

- proposal 1: 3.1%, 3.8%, 24.8%,
- proposal 2: 10.2%, 4.3%, 37.2%,
- proposal 3: 12.7%, 3.4%, 29.8%.

These results reveal an overfitting tendency of the TSA modifications.

**Figure 3:**
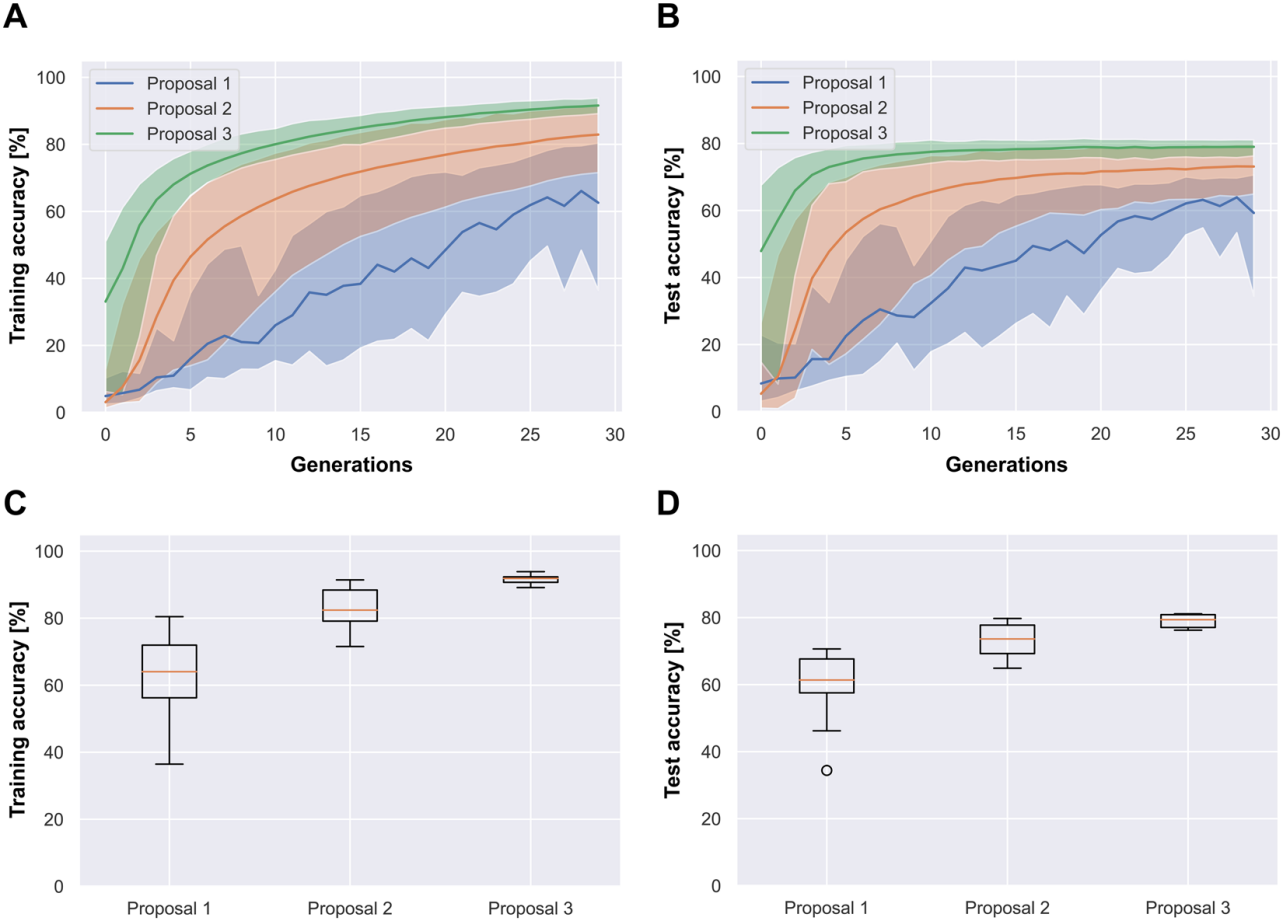
(CIFAR-100) Learning curves and boxplots for the original PathNet TSA (proposal 1), the modified TSA (proposal 2) and the modified TSA with pre-trained initialization (proposal 3). The solid lines represent the average values and the filled regions represent the minimum-to-maximum ranges.

Figure 3 shows the learning curves and box plots on the CIFAR-100 dataset. Similar results were achieved on the other datasets. The solid lines in the learning curves are the averages of the accuracies on 10 learning samples, and the filled regions delineate the ranges between the minimum and maximum values. The learning curves confirm the positive effect of the TSA modifications; namely, the learning curves of proposal 2 (green) are more accurate and stable than those of proposal 1 (blue). Furthermore, proposal 3 (red) is more accurate and stable than proposal 2, as evidenced by the smaller and more elevated filled areas on the plots. The stability trends of the three TSAs, with proposals 1 and 3 being the least and most stable respectively, are also mirrored in the boxplots.

### Comparison with other learning algorithms

**Figure 4:**
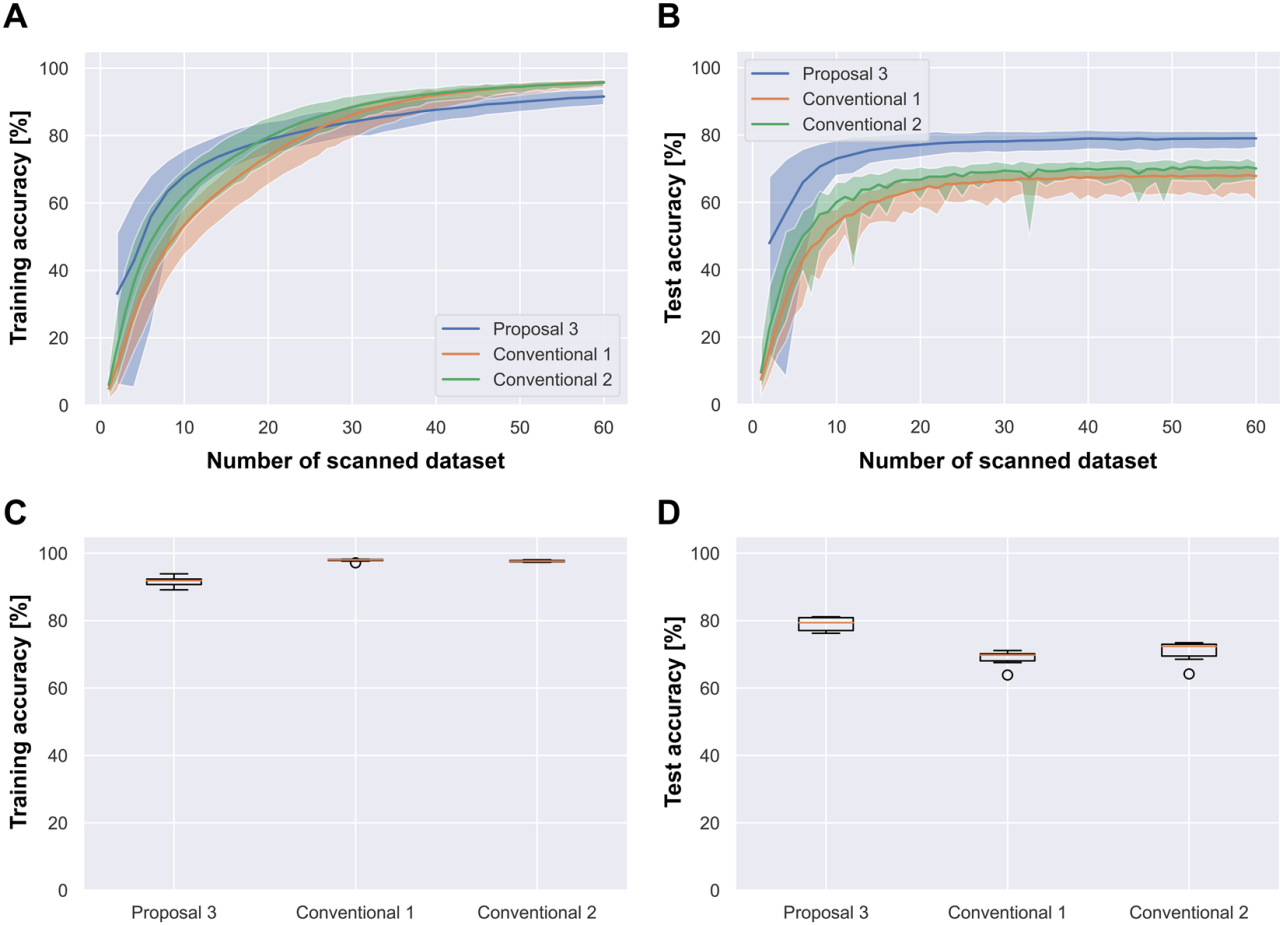
(SVHN) Comparison of learning curves and box plots for Stepwise PathNet with the modified TSA (proposal 3), the from-scratch approach (conventional 1), and fine-tuning (conventional 2).

**Figure 5:**
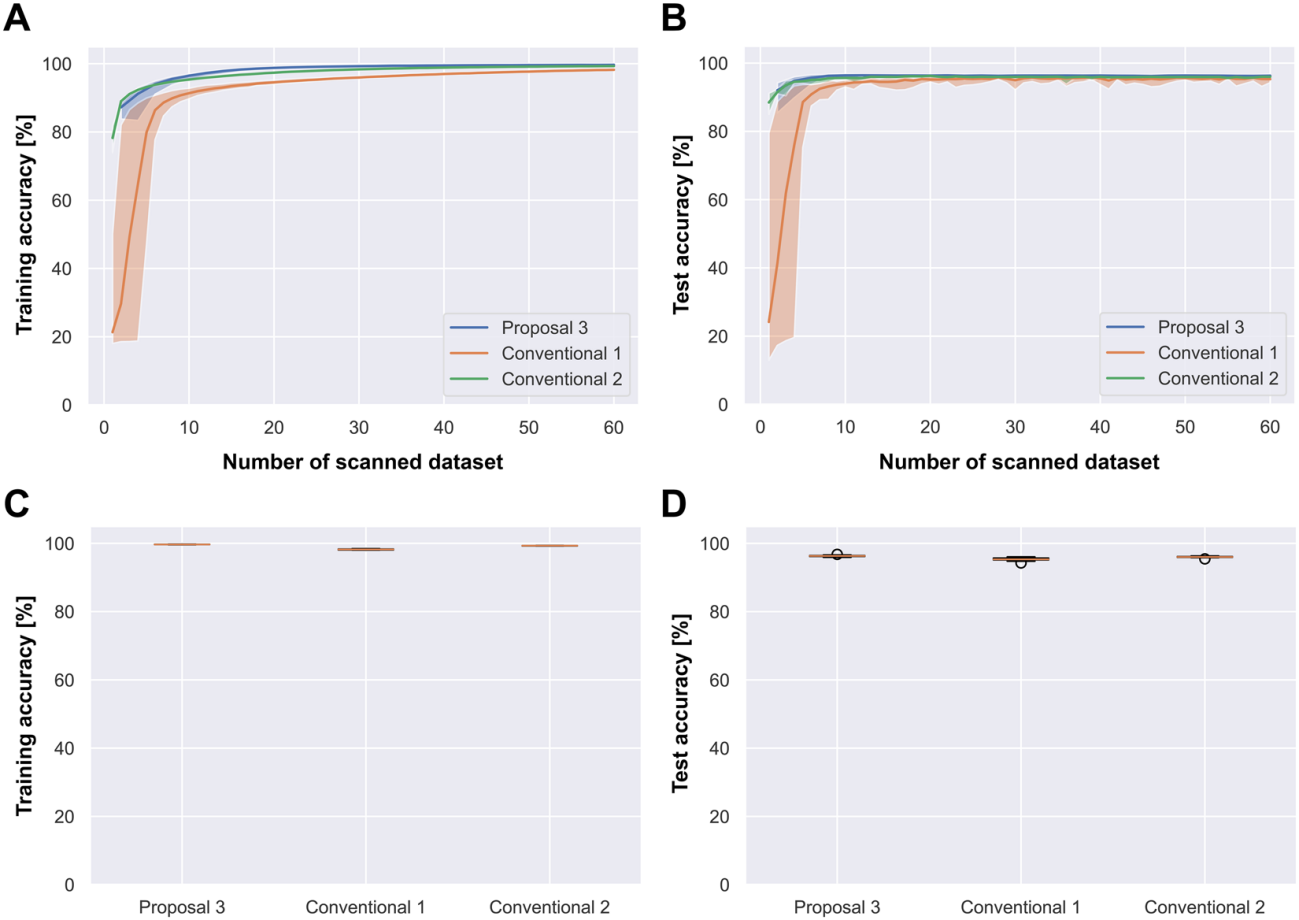
(Food-101) Comparison of learning curves and box plots for Stepwise PathNet with the modified TSA (“Ours”: blue), fine-tuning (green), and the from-scratch approach (red).

As shown in Table 1, conventional 2 outperformed conventional 1 (in terms of accuracy) on all datasets. Therefore, transfer learning from ImageNet is compatible with the CNN training except for Food-101, on which the improvement was only 0.1%. The boxplots in the bottom panels of Figs. 3 and 4 confirm that conventional 2 was more stable than conventional 1. The training accuracy of Food-101 was higher in conventional 1 than in conventional 2 (94.0% versus 92.8%), possibly because negative transfer degraded the performance of the latter. As indicated in the test-accuracy boxplot at the bottom right of Fig. 5, the instability of conventional 2 was exacerbated by proposal 3 (i.e., proposal 3 was the most unstable learning method on the Food-101 dataset). However, proposal 3 achieved the highest test accuracy among the three methods on Food-101, indicating more overfitting in this method than in the other methods.

**Figure 6:**
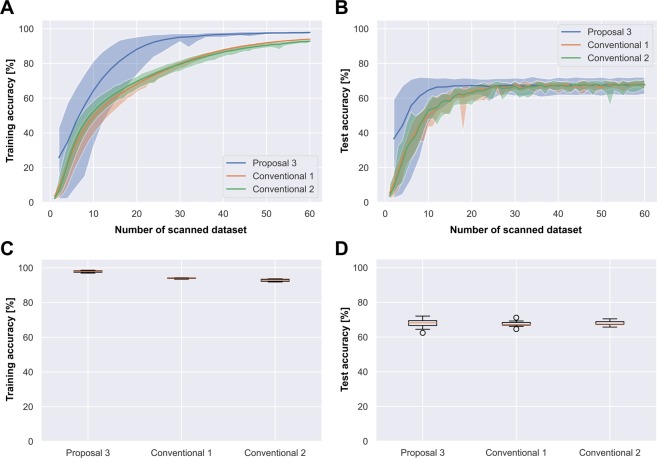
(CIFAR-100) Comparison of learning curves and box plots for Stepwise PathNet with the modified TSA (proposal 3), the from-scratch approach (conventional 1), and fine-tuning (conventional 2).

On the CIFAR-100 and SVHN datasets, proposal 3 was more accurate than from-scratch and fine-tuning. Moreover, proposal 3 better avoided the overfitting problem on CIFAR-100 than on SVHN (the most overfitted dataset, but obtaining the highest test accuracy by proposal 3). The boxplots in the right bottom panels of Figs. 4 and 6 confirm similar stabilities of the test accuracies in proposal 3 and conventional 2.

Meanwhile, the learning curves in Figs. 4, 5 and 6 show that proposal 3 converged faster than the other algorithms.

### Layer selections (geopaths)

**Figure 7:**
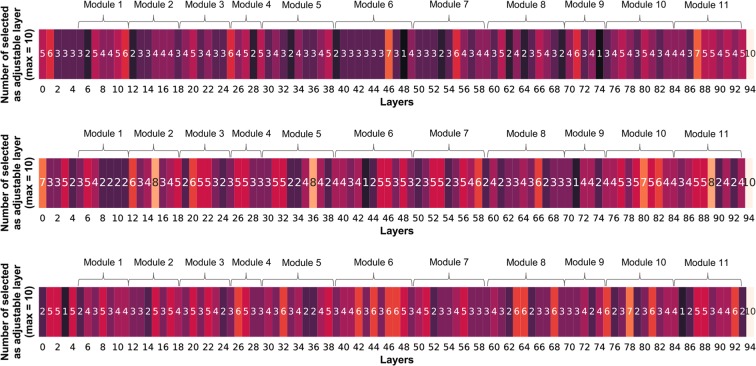
Number of times that each layer was selected as an adjustable layer among 10 transfer learnings in proposal 3. The numbers are displayed in the centers of the colored rectangles (top: CIFAR-100, middle: SVHN, and bottom: Food-101).

Figure 7 shows the heatmaps constructed for proposal 3 on the three datasets. The numbers in the colored rectangles mean the number of times that the corresponding layer was selected as an adjustable layer among the 10 transfer learnings, e.g., the first element “5” on the top heatmap means that the 0th layer of InceptionV3 was selected as an adjustable layer in five out of 10 transfer learnings from ImageNet to CIFAR-100 by proposal 3. Note that the last layer (layer 94) was always selected as an adjustable layer to ensure compatibility with the number of classes in the target task. The selection distributions do not behave like the layer function in PathNet, which tends to select the bottom and top layers as the pre-trained and adjustable layers, respectively. The heatmaps show this aberrant behavior visually.

## Discussion

### Comparison of TSAs

Proposals 1 and 2 both achieved a 96% test accuracy on the SVHN dataset, suggesting that this dataset is unsuitable for the performance comparison. The positive effect of the modification was confirmed on CIFAR-100 and Food-101, in which proposal 2 was decidedly more accurate than proposal 1. Relative to the original method (proposal 1), the TSA modification decreased the number of changes in the layer selections among the transfer-learning layers, thereby accelerating the training from the results.

Proposal 3, which initializes the adjustable layers using pre-trained weights, outperformed proposal 2. The benefit of this approach might be similar to that of fine-tuning in general CNNs. Proposal 3 adopts the same strategy as related works mentioned in the Introduction^8,14^. Combining the “fixing” and “fine-tuning” approaches also appears to deliver high performance in Stepwise PathNet. The superiority of pre-trained initialization, which is the difference between proposals 2 and 3, is attributed to the inter-layer dependence. In proposal 2, this dependence is ignored whenever an adjustable layer is selected, because the adjustable layers are initialized with random weights. However, proposal 3 usually maintains the dependence even when an adjustable layer is selected, because it is initialized with pre-trained values (at least in the first generation). The inter-layer dependence is lost only when a layer selected as a pre-trained layer was selected as an adjustable layer in the previous generation. In future work, the inter-layer dependence should be more strictly enforced for situations in which it critically affects the performance.

The source task ImageNet and CIFAR-100 are general object-recognition datasets that should be compatible with transfer learning. The Food-101 dataset, which contains images of foods on dishes, is considered as a sub-domain of general object recognition, but accurate classification results on this dataset are difficult to obtain. Therefore, we consider two cases: (i) the required information is not available in ImageNet and (ii) some information from ImageNet disturbs the training on Food-101 (negative transfer).

The overfitting on Food-101 is caused by the low compatibility between ImageNet and Food-101, as mentioned above. To untangle this problem, more evaluation of many datasets that are compatible or not compatible with ImageNet are required. Another problem is how to measure the distance (or equivalent quantity) between datasets (domains). Proposal 1 on Food-101 appears to avoid the overfitting problem, but this observation is an artefact caused by insufficient training (as evidenced by the wider variation in the training loss and accuracy than in the other algorithms). Overfitting in proposal 1 might be discussed by iterating the proposal through more generations, but the present evaluation environment lacks sufficient memory for this task.

Proposal 3 outperformed proposal 2, despite abandoning the global optimization and collapsing into a local optimum for fast convergence on the geopath searching. The superior performance of proposal 3 might be attributable to the weight parameters on the adjustable layers, which can be tuned more deeply in proposal 3 than in proposal 2. Specifically, slight differences in the selection of layers are recoverable by tuning the parameters. Therefore, the performance at convergence might not strictly depend on the layer selection. Initialization with random weights for global searching might also explain the positive effect of the TSA modification. In future work, this idea could be evaluated by tuning the TSA hyperparameters (such as the number of geopaths and number of generations).

### Comparison with other learning algorithms

The poor compatibility between ImageNet and Food-101 (as mentioned above) is also confirmed by the lower training accuracy in conventional 2 than in conventional 1. On the other hand, on CIFAR-100 and SVHN, which are considered to be compatible with ImageNet, conventional 2 achieved stable and accurate learning. When the model and augmentations are unsuitable, Food-101 is difficult to train from ImageNet data. The consequent negative transfer destabilizes the test accuracy. Proposal 2, with its randomly initialized adjustable layers, can select all layers as adjustable. In this way, it can behave similarly to the from-scratch approach, and is expected to avoid negative transfer. Unfortunately, the results confirmed that proposal 2 cannot avoid negative transfer. On the Food-101 dataset, proposal 3 outperformed proposal 2 even when negative transfer occurred. The pre-trained initialization in Stepwise PathNet is considered to benefit the learning regardless of whether the transfer is negative or positive, and is more effective for initialization (e.g., maintaining the inter-layer dependence) than pre-trained information.

A complex model with a huge number of adjustable parameters tends to be overfitted, as mentioned in the Introduction^8,9^. Proposal 3 exhibited the best overfitting avoidance on CIFAR-100, probably because selecting the pre-trained layers reduced the number of adjustable parameters. Proposal 3 adjusted total of 7.5 M parameters on average through 30 generations, while conventionals 1 and 2 adjusted total 1.3 G parameters through 60 epochs. As confirmed in the learning curves of the SVHN dataset (top panels of Fig. 4), conventional 2 and proposal 3 both achieved over 80% test accuracy, meaning that the learning better resembled re-training than transfer learning. Interestingly, despite having fewer adjustable parameters than conventional 2, proposal 3 overfitted more extensively than the conventional method. Stepwise PathNet (proposals 1-3) aims to minimize the cross-entropy and maximize the training accuracy. This probably explains why proposal 3 overfits despite the reduced number of weight parameters in re-training (or excessive epochs). More specifically, TSA can fit more even if the loss function (cross-entropy) is converged by changing the optimized geopath based on the training accuracy. It was confirmed that the variable geopath endures longest in SVHN.

As shown in the learning curves in Figs. 4–6, proposal 3 is supposed to converge to a sufficient accuracy earlier (after 30 scans) than conventional 1 and 2; however, stopping too early may destabilize the training. The filled areas in the learning curves of Stepwise PathNet were wide in the early scans (<10 scans) and narrowed as the number of scans increased. This trend, which was observed for all datasets, suggests that learning in Stepwise PathNet proceeds in two phases: (i) Optimization of the layer selection in the early scans, and (ii) fine-tuning of the weight parameters once the selection is determined to a sufficient extent. Note that these phases are not well delineated in Stepwise PathNet because they are not strictly separated in the implementation, and can change continuously. At least, if the number of generations is insufficient, the optimization is insufficient and the parameter tuning becomes confused, eventually destabilizing the training as observed in proposal 1.

### Layer selections (geopaths)

According to the theory of layer functions, the top layers are tuned while the bottom layers remain unchanged. However, this phenomenon was not observed in the present result. As mentioned above, the test accuracy did not strictly depend on the layer selection process. Of course, identifying the functions of the layers and correctly selecting the layers are maximally effective for transfer learning. However, in the case of a huge model with many layers and complicated connections, the functions of the layers are difficult to identify, and the selection becomes intractable. Although it offers only an approximate solution, the proposed Stepwise PathNet is a promising approach for handling massive networks with evolutionary behavior. Stepwise PathNet is applicable not only to CNNs but also to other neural network models (such as GANs and AutoEncoder). The potential of Stepwise PathNet needs investigating in further evaluations.

## Methods

### Related work: PathNet

#### Neural network

**Figure 8:**
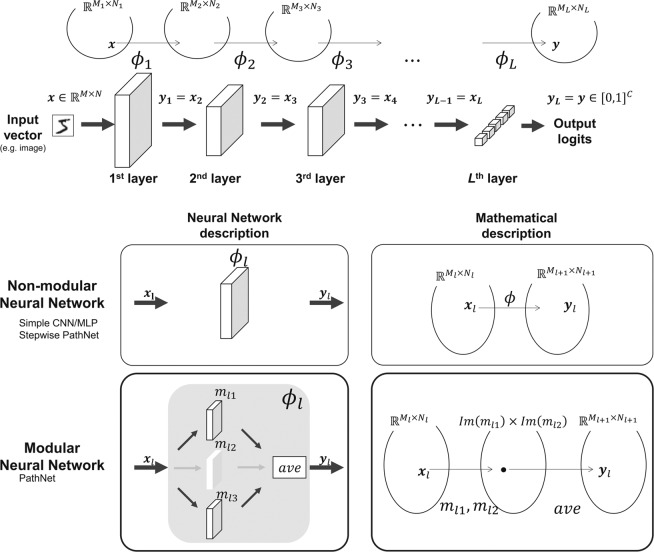
Mapping of a neural network (top), and comparison of the $l$ th layer in a simple neural network and a modular neural network (bottom).

Here, we consider an image classification task in a neural network. The neural network maps input images ${\boldsymbol{x}}\in {{\mathbb{R}}}^{M\times N}$ to output $C$-class logits ${\boldsymbol{y}}\in {[0,1]}^{C}$. The *l*th layer of the neural network (e.g., a convolutional or fully connected layer) can be expressed as the mapping

1 $${{\boldsymbol{y}}}_{l}={\phi }_{l}({{\boldsymbol{x}}}_{l}),$$

where ${{\boldsymbol{x}}}_{l}\in {{\mathbb{R}}}^{{M}_{l}\times {N}_{l}}$ and ${{\boldsymbol{y}}}_{l}\in {{\mathbb{R}}}^{{M}_{l+1}\times {N}_{l+1}}$. Iterating Eq. (1) through layers $1$ to $l$ (i.e., all layers of the neural network), a neural network with $L$ layers can be expressed as (see Fig. 8)

2 $${\boldsymbol{y}}={\phi }_{L}({\phi }_{L-1}(\cdots {\phi }_{l}(\cdots ({\phi }_{2}({\phi }_{1}({\boldsymbol{x}}))))))$$

3 $$\,={\phi }_{L}\circ \cdots \circ {\phi }_{2}\circ {\phi }_{1}({\boldsymbol{x}})=\Phi ({\boldsymbol{x}}\mathrm{.)}$$

The training dataset ${\mathcal{D}}$ is expressed as the following set of pairs:

4 $${\mathcal{D}}\subset \{({\boldsymbol{x}},{{\boldsymbol{y}}}^{(t)})|{\boldsymbol{x}}\in {{\mathbb{R}}}^{M\times N},{{\boldsymbol{y}}}^{(t)}\in {[0,1]}^{C},\mathop{\sum }\limits_{i=1}^{C}\,{y}_{i}^{(t)}=1\},$$

where ***x*** is an input image and ***y***^(*t*)^ is a teacher signal (label), required for calculating the cross-entropy loss function

5 $$H({{\boldsymbol{y}}}^{(t)},\Phi ({\boldsymbol{x}}))=H({{\boldsymbol{y}}}^{(t)},{\boldsymbol{y}})=-\,\mathop{\sum }\limits_{i=1}^{C}\,{y}_{i}\,\log \,{y}_{i}^{(t)}\mathrm{}.$$

This function measures the distance $H()$ between ${\boldsymbol{y}}=\Phi ({\boldsymbol{x}})$ and ${{\boldsymbol{y}}}^{(t)}$. The learning process of the neural network corresponds to solving an optimization problem that tunes the mappings $\{{\phi }_{l}:l=1,2,\ldots ,L\}$ to minimize the sum of the loss functions in dataset ${\mathcal{D}}$:

6 $$\sum _{({\boldsymbol{x}},{{\boldsymbol{y}}}^{)t)})\in {\mathcal{D}}}\,H({{\boldsymbol{y}}}^{(t)},\Phi ({\boldsymbol{x}}\mathrm{))}.$$

#### Modular neural network

PathNet is based on a modular neural network composed of modules (Fig. 1). The set of modules ${{\mathcal{M}}}_{l}$ in the $l$ th layer of PathNet is defined as

7 $${{\mathcal{M}}}_{l}\subset \{m|m:{{\mathbb{R}}}^{{M}_{l}\times {N}_{l}}\to {{\mathbb{R}}}^{{M}_{l+1}\times {N}_{l+1}}\},$$

where $|{{\mathcal{M}}}_{l}|$ (i.e., the cardinality of ${{\mathcal{M}}}_{l}$) is the number of modules. Each module $m$ is configurable by the user. Note that only some of the modules in ${{\mathcal{M}}{\prime} }_{l}$ are used. The set of used modules (called active modules) is a subset of ${{\mathcal{M}}}_{l}$:

8 $${{\mathcal{M}}{\prime} }_{l}\subset {{\mathcal{M}}}_{l}\mathrm{}.$$

Note that the number of active modules $|{{\mathcal{M}}{\prime} }_{l}|$ is limited to $|{{\mathcal{M}}{\prime} }_{l}| < {\mu }_{l}$, where ${\mu }_{l}$ is a configurable hyperparameter. In the example given in the lower left panel of Fig. 8, ${m}_{l2}$ is a non-active module, whereas ${m}_{l1}$ and ${m}_{l3}$ are active modules. Therefore, we have ${{\mathcal{M}}}_{l}=\{{m}_{l1},{m}_{l2},{m}_{l3}\}$, and ${{\mathcal{M}}{\prime} }_{l}=\{{m}_{l1},{m}_{l3}\}$. The mapping of the $l$ th layer in the modular neural network

9 $${\phi }_{l}:{{\mathbb{R}}}^{{M}_{l}\times {N}_{l}}\to {{\mathbb{R}}}^{{M}_{l+1}\times {N}_{l+1}}$$

can be expressed as

10 $${{\boldsymbol{y}}}_{l}={\phi }_{l}({{\boldsymbol{x}}}_{l})=\frac{1}{|{{\mathcal{M}}{\prime} }_{l}|}\,\sum _{m\in {{\mathcal{M}}{\prime} }_{l}}\,m({{\boldsymbol{x}}}_{l}).$$

A module $m(\cdot )$ is a tiny layer such as a simple perceptron $fsp$, skip layer ${f}_{skip}$, or residual layer ${f}_{res}$, respectively expressed as:

11 $${f}_{sp}=\{f({{\boldsymbol{x}}}_{l})=act({{\boldsymbol{W}}}_{ml}\cdot {{\boldsymbol{x}}}_{l}+{{\boldsymbol{b}}}_{ml})\}$$

12 $${f}_{skip}=\{f({{\boldsymbol{x}}}_{l})={{\boldsymbol{W}}}_{ml}\cdot {{\boldsymbol{x}}}_{l}+{{\boldsymbol{b}}}_{ml}\}$$

13 $${f}_{res}=\{f({{\boldsymbol{x}}}_{l})=act({{\boldsymbol{W}}}_{ml}\cdot {{\boldsymbol{x}}}_{l}+{{\boldsymbol{b}}}_{ml})+{{\boldsymbol{x}}}_{l}\}$$

Therefore, ${{\mathcal{M}}}_{l}$ can also be expressed as

14 $${{\mathcal{M}}}_{l}\subset \{m|m\in {f}_{sp}\cup {f}_{rnn}\cup {f}_{skip}\}\}\subset \{m|m:{{\mathbb{R}}}^{{M}_{l}\times {N}_{l}}\to {{\mathbb{R}}}^{{M}_{l+1}\times {N}_{l+1}}\mathrm{\}}.$$

As the activation function $act()$, we adopt the rectified linear unit (ReLU)^25^, expressed as

15 $$act(x)=\{\begin{array}{ll}0 & {\rm{if}}\,x < 0\\ x & {\rm{if}}\,x\ge 0.\end{array}$$

#### Tournament selection algorithm

A modular neural network is learned using the TSA^15^, which is based on the microbial genetic algorithm^16^. The dual objectives are to minimize the loss function and maximize the accuracy by optimizing the active modules. An $L$-layer modular neural network is expressed by sets of active modules referring to ${{\mathcal{M}}{\prime} }_{l}$, namely

16 $${\mathcal{M}}=\{{{\mathcal{M}}{\prime} }_{l}:l=1,2,\ldots ,L\}.$$

A geopath $G$ is a set of gene expressions such as

17 $$G=\{{{\boldsymbol{g}}}_{l}:l=1,2,\ldots L\},$$

where ${{\boldsymbol{g}}}_{l}\in {\{0,1\}}^{|{{\mathcal{M}}}_{l}|}$ expresses the inactive ($0$) and active ($1$) modules in the $l$ th layer. In the example given in the bottom left panel in Fig. 8, module ${m}_{l2}$ is inactive, whereas ${m}_{l1}$ and ${m}_{l3}$ are active, giving ${{\boldsymbol{g}}}_{l}=\{1,0,1\}$.

In the initialization step, the $P$ geopaths expressed in Eq. (17) are generated randomly. Then

18 $${{\mathfrak{G}}}^{(t)}=\{{G}_{1},{G}_{2},\ldots ,{G}_{P}\}$$

is defined as a set of geopaths at epoch (generation) $t=0$.

Additionally, the set of all modules in the *t*th generation is taken as the set of selectable modules in the *l*th layer ${M}_{l}(l=1,2,\ldots ,L)$, namely

19 $${{\mathfrak{M}}}^{(t)}=\{{M}_{1},{M}_{2},\ldots ,{M}_{L}\}.$$

The *i*th module in the *l*th layer is defined as

20 $${m}_{li}\in {M}_{l}\subset {{\mathfrak{M}}}^{(t)}.$$

For simplicity, this module is sometimes written as ${m}_{li}\in {{\mathfrak{M}}}^{(t)}$. The weight of ${m}_{li}$, which is a small layer in the neural network, is initialized by Eq. (11). The initialization is performed with a truncated normal and a constant, as in non-modular neural networks.

In the learning process, two geopaths are randomly selected from ${{\mathfrak{G}}}^{(t)}$ as follows:

21 $${G{\prime} }_{1},{G{\prime} }_{2}\in {{\mathfrak{G}}}^{(t)}.$$

Referring to Eqs. (4) and (6), the cross entropies are summed as

22 $${F}_{1}(G)=\sum _{({\boldsymbol{x}},{{\boldsymbol{y}}}^{(t)})\in {\mathcal{D}}}\,H({{\boldsymbol{y}}}^{(t)},\Phi ({\boldsymbol{x}})),$$

where $\Phi$ is the neural network corresponding to $G$. This sum is employed as the loss function for learning ${G{\prime} }_{1},{G{\prime} }_{2}$. During the learning process, Eq. (22) is minimized by the SGD method. The accuracy of determining ${G}_{win}^{(t)}\,and\,{G}_{lost}^{(t)}$ is then measured as

23 $${F}_{2}(G)=\frac{|\{({\boldsymbol{x}},{{\boldsymbol{y}}}^{(t)})\in {\mathcal{D}}|{\rm{\arg }}\,{\rm{\max }}\,{{\boldsymbol{y}}}^{(t)}={\rm{\arg }}\,{\rm{\max }}\,\Phi ({\boldsymbol{x}})\}|}{|{\mathcal{D}}|}.$$

If ${F}_{2}({G{\prime} }_{1}) > {F}_{2}({G{\prime} }_{2})$, then ${G}_{win}^{(t)}\,and\,{G}_{lost}^{(t)}$ are set as

24 $${G}_{win}^{(t)}={G{\prime} }_{1},\,{G}_{lost}^{(t)}={G{\prime} }_{2},$$

and ${G}_{lost}^{(t)}$ is overwritten and mutated. The set of geopaths

25 $${{\mathfrak{G}}}^{(t+1)}=\{{G}_{win}^{(t)},{G}_{lost}^{(t)}\},\cup ({{\mathfrak{G}}}^{(t)}\backslash \{{G{\prime} }_{1},{G{\prime} }_{2}\})$$

is then updated at epoch $t+1$.

The weight updates preferentially update the weights of the winning modules.

Defining the modular neural networks corresponding to ${G}_{win}^{(t)}$ and ${G}_{loss}^{(t)}$ as ${{\mathcal{M}}}_{win}^{(t)}$ and ${{\mathcal{M}}}_{loss}^{(t)}$ respectively, ${{\mathcal{M}}}^{(t+\mathrm{1)}}$ is updated as

26 $${m}_{li}\in {{\mathcal{M}}}_{win}^{(t)}\cup ({{\mathcal{M}}}_{loss}^{(t)}\backslash {{\mathcal{M}}}_{win}^{(t)}).$$

The modular neural network is learned by repeating the above learning process for $t=1,2,\ldots ,T$.

#### Transfer learning using PathNet

Transfer learning reduces the size of a training dataset by utilizing the knowledge in a pre-trained neural network. Transfer learning accomplishes two tasks: (i) the source task, which is learned by the pre-trained neural network, and (ii) the target task, which is performed on the training dataset. Here, we refer to the datasets of the source and target tasks as ${{\mathcal{D}}}^{(S)}$ and ${{\mathcal{D}}}^{(T)}$, respectively, and define transfer learning as a learning method for the target task using a pre-trained neural network learned for the source task.

The modular neural networks of the source and target tasks in Eq. (16) are denoted as ${{\mathcal{M}}}^{(S)}$ and ${{\mathcal{M}}}^{(T)}$, respectively. Transfer learning using PathNet constructs a modular neural network ${{\mathcal{M}}}^{(T)}$ for the target task using the modular neural network ${{\mathcal{M}}}^{(S)}$ pre-trained on the dataset ${{\mathcal{D}}}^{(S)}$ of the source task using the TSA. Here, ${{\mathcal{M}}}^{(S)}$ and ${{\mathcal{M}}}^{(T)}$ have the same structure, i.e., the same number of layers and the same modules in each layer $l$. Therefore, the active modules of the source task ${{\mathcal{M}}}^{(S)}$ and target task ${{\mathcal{M}}}^{(T)}$ are subsets of ${{\mathcal{M}}}_{l}$, expressed as

27 $${{\mathcal{M}}{\prime} }_{l}^{(S)},\,{{\mathcal{M}}{\prime} }_{l}^{(T)}\subset {{\mathcal{M}}}_{l}\mathrm{}.$$

In the learning process of the target task, when a module of the source task

28 $$m\in {{\mathcal{M}}{\prime} }_{l}^{(S)}\cap {{\mathcal{M}}{\prime} }_{l}^{(T)}$$

is selected as an active module, its parameters are fixed during the SGD method.

In some transfer learning cases, the pre-trained neural network for the source task has been trained by a large computer. The pre-trained neural networks on the Internet are non-modular neural networks. Therefore, PathNet requires a mapping $\psi$ between Eqs. (1) and (10); for instance,

$$\psi :\{\phi |\phi :{{\mathbb{R}}}^{{M}_{l}\times {N}_{l}}\to {{\mathbb{R}}}^{{M}_{l+1}\times {N}_{l+1}}\}\to \{{\mathcal{M}}|{\mathcal{M}}\subset \{m|m:{{\mathbb{R}}}^{{M}_{l}\times {N}_{l}}\to {{\mathbb{R}}}^{{M}_{l+1}\times {N}_{l+1}}\}\}.$$

This expression shows that PathNet can extend the versatility of network structures.

### Proposed method: Stepwise PathNet

As mentioned in the previous subsection, the versatility of PathNet can be improved by relaxing the restrictions on the transfer learning processes, namely, that ${{\mathcal{M}}}^{(S)}$ and ${{\mathcal{M}}}^{(T)}$ have the same structure. This paper proposes Stepwise PathNet as an extension of PathNet. The proposed Stepwise PathNet regards each layer as a module.

Following Eq. (3), a pre-trained neural network is given as

$${\boldsymbol{y}}={\phi }_{L}^{(S)}\circ \cdots \circ {\phi }_{l}^{(S)}\circ \cdots \circ {\phi }_{2}^{(S)}\circ {\phi }_{1}^{(S)}({\boldsymbol{x}})={\Phi }^{(S)}({\boldsymbol{x}}).$$

In Stepwise PathNet, a layer is specified as the following module:

29 $${\phi }_{l}^{(S)}({{\boldsymbol{x}}}_{l})={m}_{l}^{(S)}({{\boldsymbol{x}}}_{l}).$$

The *l*th layer of the neural network ${{\mathcal{M}}}^{(T)}$ of the target task is defined as

30 $${{\mathcal{M}}}_{l}^{(T)}=\{{m}_{l}^{(S)},{m}_{l}\}\subset \{m|m:{{\mathbb{R}}}^{{M}_{l}\times {N}_{l}}\to {{\mathbb{R}}}^{{M}_{l+1}\times {N}_{l+1}}\},$$

where ${m}_{l}^{(S)}$ is the pre-trained (with a fixed parameter) layer, and ${m}_{l}$ is a layer with an adjustable parameter (see Fig. 1).

The set of active modules ${{\mathcal{M}}{\prime} }_{l}^{(T)}\subset {{\mathcal{M}}}_{l}^{(T)}$ includes either ${m}_{l}^{(S)}$ or ${m}_{l}$:

31 $${{\mathcal{M}}{\prime} }_{l}^{(T)}=\{{m}_{l}^{(T)}\}=\{{\phi }_{l}^{(T)}\}.$$

As $|{{\mathcal{M}}{\prime} }_{l}^{(T)}|=1$, we have

32 $${{\boldsymbol{y}}}_{l}={\phi }_{l}^{(T)}({{\boldsymbol{x}}}_{l})={m}_{l}^{(T)}({{\boldsymbol{x}}}_{l}).$$

Therefore, the non-modular neural network

33 $${\boldsymbol{y}}={\phi }_{L}^{(T)}\circ {\phi }_{L-1}^{(T)}\circ \cdots \circ {\phi }_{l}^{(T)}\circ \cdots \circ {\phi }_{2}^{(T)}\circ {\phi }_{1}^{(T)}({\boldsymbol{x}}),={\Phi }^{(T)}({\boldsymbol{x}}),$$

can be constructed by the proposed Stepwise PathNet. The proposed method removes the need for mapping $\psi$ in Eq. (29). This relaxation is the contribution of Stepwise PathNet to the existing arsenal of neural network methods.

We now introduce an improved version of TSA () for use in Stepwise PathNet.

The proposed TSA differs from PathNet’s original algorithm in two aspects: (i) the initialization of the geopath and (ii) use of a selection method in the learning process in each epoch. When initializing the geopaths $p=1,2,\ldots ,P$, the conventional method randomly selects ${m}_{l}^{(S)}$ or ${m}_{l}$, whereas the proposed method randomly selects ${m}_{l}^{(S)}$ or ${m}_{l}$ after assigning the following weights:

- ${w}_{p}$: weight of selecting the pre-trained layer ${m}_{l}^{(S)}$
- ${w{\prime} }_{p}$: weight of selecting the adjustable layer ${m}_{l}$,

where ${w}_{p}+{w{\prime} }_{p}=1$. In addition, the weight ${w}_{p}$ of the *p*th geopath is given as

34 $${w}_{p}=\frac{p-1}{P-1}.$$

These weights control the tendency of the layer selection at each geopath initialization. If ${w}_{p} > {w}_{p}^{{\prime} }$, the *p*th geopath initialization tends to select a pre-trained layer. This initialization varies the geopaths, enabling a more global search process. In other words, the proposed initialization method can seek an optimal solution more effectively than the original initialization. During the learning process in each epoch, the original method randomly selects two geopaths, whereas the proposed method uses the winning geopath in the previous epoch and one randomly selected geopath:

35 $${G{\prime} }_{1}={G}_{win}^{(t-1)}\in {{\mathfrak{G}}}^{t-1},\,{G{\prime} }_{2}\in {{\mathfrak{G}}}^{t}.$$

This selection method stabilizes and accelerates the learning process. The previous winning geopath can be overwritten only by a higher-scoring geopath, yielding a nearly monotonic increase in the learning curve and a speedy geopath convergence. At the $t=0$ th epoch, the previous winning geopath is given as ${G}_{win}^{(t-1)}={G}_{win}^{(-1)}$; for example,

36 $${G}_{win}^{(-1)}={G}_{0}=\{(0),(0),\ldots ,(0)\}.$$

Here, ${G}_{win}^{(-\mathrm{1)}}$ has only an adjustable layer, implying from- scratch training without transfer learning. Therefore, the learned geopath is expected to be more accurate than one learned from scratch.

## Conclusion

We proposed a new transfer learning algorithm (Stepwise PathNet) that addresses the problem of layer selection in CNN transfer learning. We also modified the TSA learning algorithm for Stepwise PathNet. The modified TSA and initialization of the adjustable layers with pre-trained values were experimentally evaluated in transfer learning from ImageNet learned by the InceptionV3 image classifier to three datasets (CIFAR-100, SVHN and Food-101) learned by Stepwise PathNet. By modifying the TSA and using pre-trained values in the adjustable layer, we achieved more stable and faster transfer learning than was possible with the original TSA and random initialization. Moreover, Stepwise PathNet with the modified TSA and pre-trained values outperformed both the fine-tuning and from-scratch approaches (improving the average test accuracies by up to 8% and 10%, respectively), and its performance was largely independent of the layer selection. In future work, we will aim to (i) analyze and improve the stability of genetic-algorithm-based methods, (ii) analyze the layer-selection process, and (iii) investigate other domain settings, including difficult transfer-learning scenarios.
